# Supplementary material for: How a Highly Acidic SH3 Domain Binds to Its Intrinsically Disordered Partner through the Formation of an Encounter Complex Intermediate
Source: J Phys Chem B. 2026 Oct 5;130(41):10855–72. doi: 10.1021/acs.jpcb.6c05098 (PMC13679038; doi:10.1021/acs.jpcb.6c05098)
Supplement: Supplementary file 2 [file jp6c05098_si_002.pdf]

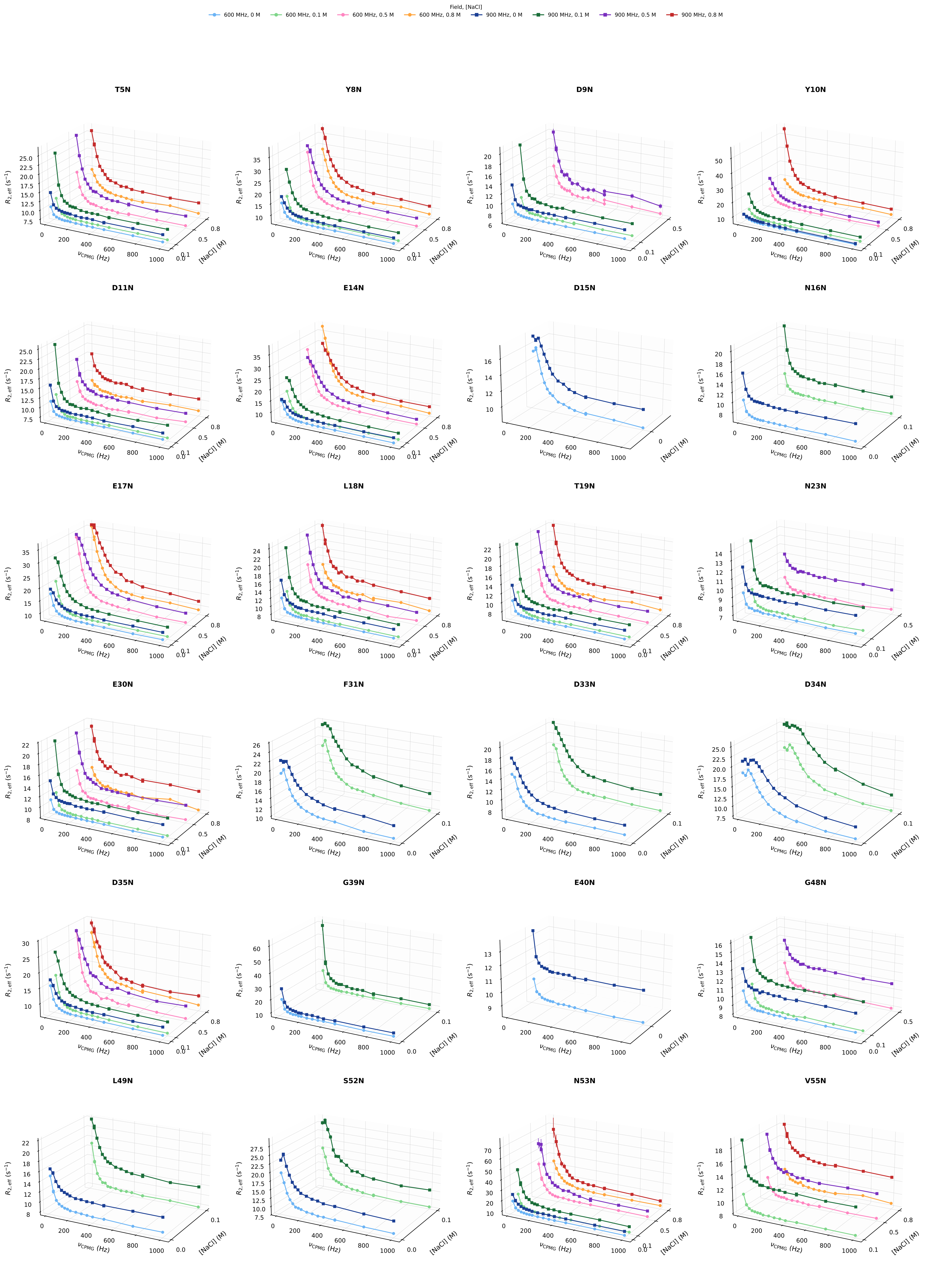
Field, [NaCl]
600 MHz, 0 M
600 MHz, 0.1 M
600 MHz, 0.5 M
600 MHz, 0.8 M
900 MHz, 0 M
900 MHz, 0.1 M
900 MHz, 0.5 M
900 MHz, 0.8 M
T5N
Y8N
D9N
Y10N
D11N
E14N
D15N
N16N
E17N
L18N
T19N
N23N
E30N
F31N
D33N
D34N
D35N
G39N
E40N
G48N
L49N
S52N
N53N
V55N
$R_{2,eff}$ (s^{-1})
ν_{CPMG} (Hz)
[NaCl] (M)
